# Changes in Microbiota Composition Along the Metamorphosis Developmental Stages of *Chironomus transvaalensis*

**DOI:** 10.3389/fmicb.2020.586678

**Published:** 2020-11-06

**Authors:** Rotem Sela, Sivan Laviad-Shitrit, Malka Halpern

**Affiliations:** ^1^Department of Evolutionary and Environmental Biology, Faculty of Natural Sciences, University of Haifa, Haifa, Israel; ^2^Department of Biology and Environment, Faculty of Natural Sciences, University of Haifa, Tivon, Israel

**Keywords:** chironomid, microbiome, host–bacteria interaction, insect, *Vibrio*, *Aeromonas, Deinococcus-Thermus*

## Abstract

Chironomids (*Diptera*; *Chironomidae*), also known as non-biting midges, are one of the most abundant insects in freshwater habitats. Our aim was to understand whether the metamorphosis developmental stages affect the endogenous microbiota composition of *Chironomus transvaalensis*. Toward our objective, we analyzed the endogenous microbiota composition of *C. transvaalensis*’ four life stages: egg masses, larvae, pupae, and adults. Significant differences were found between the microbiota compositions of the different developmental stages of this *Chironomus* species. We observed a decline in bacterial diversity as the insect evolved from egg mass to adult, while the highest richness was observed in the pupal stage. Although there were significant differences between the microbiota compositions of each life stage, a bacterial core, which included 27 Amplicon Sequence Variants (ASVs), was found in all the developmental life stages (in ≥75% of samples). Chironomids are natural reservoirs of *Vibrio cholerae* and *Aeromonas* species, and the *Vibrio* and *Aeromonas* ASVs were part of the core bacteria. The presence of the *ompW* gene, which is specific to *V. cholerae*, confirmed the presence of this species in all the chironomid’s life stages. Thus, the results provide important insights about the host–microbe interactions in chironomids with a specific understanding of chironomids-*Vibrio-Aeromonas*-microbiota interactions.

## Introduction

Chironomids are the most widely dispersed aquatic insects in freshwater habitats (Ferrington, 2008). They can tolerate extreme temperature, pH, salinity, depth, and current velocity, and even dehydration and UV and gamma radiation (Ali, 1991; Senderovich and Halpern, 2012, 2013; Thorat and Nath, 2015, 2016). Chironomids are an important component of the food chain as a food source for invertebrates, fish, and birds (Floss et al., 2013).

Chironomids undergo a complete metamorphosis of four life stages. Eggs, larvae, and pupae occur in water, and adults emerge into the air. Females deposit hundreds of eggs that float in jelly-like masses. These egg masses are embedded in a gelatinous matrix that is composed of glycoprotein and chitin (Halpern et al., 2003; Laviad et al., 2016). The first instar larva feeds on the egg mass remnants and then swims toward the bottom of the water habitat, where it constructs a silken tube (Thorat and Nath, 2018). The larva passes through four instars before it transforms into a pupa, from which a flying adult emerges into the air (Armitage et al., 1995; Thorat and Nath, 2013). The adults do not feed and only live for a short period to mate and lay eggs. Adult males create aerial mating swarms after which the females lay egg masses at the water’s edge.

To date, few studies were performed on the bacterial communities associated with chironomid egg masses and larvae, and to the best of our knowledge, no data have been published regarding pupa or adult microbiota composition (Halpern and Senderovich, 2015). Rouf and Rigney (1993), Halpern et al. (2007), and Lotfi et al. (2016) studied the egg mass microbiota composition using culturable methods, whereas Sela and Halpern (2019) used Illumina sequencing of the 16S rRNA gene to study the egg mass microbiota composition and dynamics along 1 year of sampling. Two studies (Senderovich and Halpern, 2012, 2013) examined a few egg mass and larval bacterial community compositions, and using 454-pyrosequencing of the 16S rRNA gene, Senderovich and Halpern (2013) found significant differences between these communities. The microbiota associated with egg masses was more diverse compared to those associated with larvae. Further, they demonstrated that the larval microbiota plays a role in protecting the insect from toxic metals.

*Vibrio cholerae* is the causative agent of the diarrheal disease cholera that emerges in outbreaks such as the one in Haiti that followed the 2010 earthquake (Chin et al., 2011). *V. cholerae* is a natural inhabitant of aquatic ecosystems and is commonly isolated in samples from all four chironomid life stages (Broza and Halpern, 2001; Halpern and Senderovich, 2015). *Aeromonas* species cause diseases in invertebrates and vertebrates (like fish and birds) and are also responsible for gastroenteritis and wound infection in humans (Laviad and Halpern, 2016). Various *Aeromonas* species were isolated and identified from chironomid egg masses and in the larval stage (Senderovich et al., 2008; Beaz-Hidalgo et al., 2012; Halpern and Senderovich, 2015; Laviad and Halpern, 2016).

Our aim was to understand if the metamorphosis affects the endogenous microbiota composition of *Chironomus transvaalensis*. As far as we know, this has never been studied before for any of the chironomid species. Our results demonstrate that there are significant differences between the microbiota compositions of the different *C. transvaalensis* life stages, which improves our understanding of chironomid-*Vibrio-Aeromonas*-microbiota interactions.

## Materials and Methods

### Chironomid Sampling Site

All chironomid samples were collected on July 19, 2018, at a waste stabilization pond in Northern Israel (longitude 35.1245, latitude 32.6582). In total, 32 samples that were identified as belonging to the same chironomid species (*C. transvaalensis*) were selected for further analyses. Of these, ten were egg masses, seven larvae, nine pupae, and six adults (Table 1).

**Table 1:** Molecular detection of *Vibrio cholerae* (*ompW* positive), in the different *Chironomus transvaalensis* life stage samples.

Life stage	*n*	*ompW* (Number of positive samples)
Egg mass	10	8
Larva	7	5
Pupa	9	7
Adult	6	5

### Chironomid Sampling Procedure

Egg masses, larvae, pupae, and adults were collected directly from plants and sediments in the waste stabilization pond, and then samples were immediately delivered to the laboratory. Each sample was washed and vortexed individually in a sterile saline solution, for 1 min. This washing procedure was repeated five times and resulted in the removal of surface contaminants and any bacteria that was not firmly attached to the samples. Samples were kept in 2 ml 95% ethanol at −20°C until further analysis.

### DNA Extraction

To remove ethanol residues, each sample was centrifuged for 30 min at maximum speed, and then tubes were heated in a heat block for 10 min at 100°C. Each specimen was washed and homogenized separately in sterile saline water using a sterile homogenizer. DNA was extracted using a DNA isolation kit (DNeasy Blood and Tissue, Qiagen, Germany), according to the manufacturer’s instructions with minor adjustments, as was described previously (Laviad-Shitrit et al., 2017). Briefly, 20 mg/ml lysozyme (from chicken egg white, Sigma-Aldrich, United States) and 180 μl sterile enzymatic lysis buffer (20 mM Tris HCl pH 8, 2 mM sodium EDTA, and 1.2% Triton-X-100) were added to each sample, and samples were then incubated with shaking for 60 min at 37°C. Next, 25 μl proteinase K and 200 μl Al buffer from the DNeasy kit were added to each sample. Samples were incubated with shaking for 30 min at 56°C. No DNA was detected when extractions were performed on three blanks without addition of a chironomid sample. The extracted DNA was stored at −20°C.

### Chironomids’ Species Identification

The taxonomic identification of all samples was performed by PCR amplification and sequencing of the cytochrome oxidase subunit I gene, according to Edgar et al. (2011). Each 25 μl PCR reaction consisted of 10 μl DNA extract, 10 μl Takara Master Mix (Clontech, Japan), 1 μl of each primer, F-911 (5'-TTTCTACAAATCATAAAGATATTGG-3'), and R-912 (5'-TAAACTTCAGGGTGACCAAAAAATCA-3'; Sigma-Aldrich, Israel), at a concentration of 10 ng/μl, and 3 μl H_2_O (Carew et al., 2003; Moonsamy et al., 2013). The reaction conditions were as described by Folmer et al. (1994). Amplicons were sequenced by MCLAB laboratories (San Francisco, CA, United States) and were analyzed using the National Center for Biotechnology Information (NCBI) BLAST engine. Sequences were deposited in the NCBI GenBank database under the accession numbers MN934105-MN934150.

### Molecular Identification of *Vibrio cholerae*

The presence of *V. cholerae* in *C. transvaalensis* samples was detected using primers for the *ompW* gene (an outer membrane protein specific to *V. cholerae*) according to Nandi et al. (2000).

### Microbial Community Characterization With 16S rRNA Gene Amplicon Sequencing

Genomic DNA was prepared for sequencing using a two-stage amplicon sequencing workflow, as described previously (Naqib et al., 2018). Initially, genomic DNA was PCR amplified using primers targeting the V4 region of microbial 16S ribosomal RNA (rRNA) genes. The primers, 515F and 806R, were synthesized with 5' linker sequences compatible with Access Array primers for Illumina sequencers (Caporaso et al., 2012; Sigma-Aldridge, Israel): CS1_515F: ACACTGACGACATGGTT CTACAGTGCCAGCMGCCGCGGTAA and CS2_806R: TACGGTAGCAGAGACTTGGTCTGGACTACHVGGGTWTC TAAT-3'. PCR amplification was performed as described by Sela and Halpern (2019). Briefly, amplification was performed using Takara Master Mix (Clontech, Japan) with a final primer concentration of 0.5 ng/μl for each primer. Genomic DNA in the amount of 10–100 ng was added to each PCR reaction. Thermocycling conditions were as follows: 95°C for 5 min, followed by 28 cycles of 30 s at 95°C; 45 s at 55°C, and 30 s at 68°C and a final elongation step of 7 min at 68°C. The amplified PCR products were approximately 336 bp long (the PCR product without linkers is 292–293 bp, and the CS linkers are 22 bp each). Prior to storage at −20°C, amplicon size was verified by agarose gel electrophoresis. Sterile DNA free H_2_O was used as a negative control to verify the absence of contamination in this process, and no visible contamination was observed.

To incorporate Illumina sequencing adapters and a sample-specific barcode, a second PCR amplification was performed in 10 μl reactions in 96-well plates using the MyTaq HS 2× master mix (Bioline, London, UK). Each well received a separate primer pair with a unique 10-base barcode, obtained from the Access Array Barcode Library for Illumina (Item# 100-4876, Fluidigm, South San Francisco, CA). These Access Array primers, which contain Illumina adapters and sample-specific barcodes, also contain the CS1 and CS2 linkers at the 3' ends of the oligonucleotides. Thermocycling conditions were: 95°C for 5 min, followed by 8 cycles of 95°C for 30 s, 60°C for 30 s, and 72°C for 30 s. Barcoded amplicons were pooled in equal volume using an EpMotion5075 liquid handling robot (Eppendorf, Hamburg, Germany). The pooled library was purified using the AMPure XP cleanup protocol (0.6X, vol/vol; Agencourt, Beckmann-Coulter) to remove fragments smaller than 300 bp. The pooled libraries, with a 20% phiX spike-in, were loaded onto an Illumina MiniSeq mid-output flow cell employing paired-end 2 × 150 base reads. Based on the distribution of reads per barcode, the amplicons (prior to purification) were re-pooled to generate a more balanced distribution of reads. The re-pooled library was purified by AMPure XP cleanup and again loaded onto a Miniseq flow cell and sequenced (2 × 150 base paired-end reads). Fluidigm sequencing primers, targeting the CS1, and CS2 linker regions were used to initiate sequencing. De-multiplexing of reads was performed on the instrument. Second-stage PCR amplification, pooling, and sequencing were performed at the Genome Research Core (GRC) at the University of Illinois at Chicago (UIC).

### Sequence Analysis

In total, 128 files in fastq format were generated, corresponding to 32 samples (four files for each sample), two pair-ends sequenced each. Data were examined with the fastQC program,^1^ which provides a series of detailed statistics and plots for each individual fastq. All the samples were of high quality in both directions of sequencing. Bioinformatic analysis was performed with the DADA2 pipeline (DADA2 v.1.14.0; Callahan et al., 2016). Raw sequences were quality filtered, trimmed (maxN = 0, maxEE = 2, trimLeft = 15 bp, and truncLen = 150), and merged (min overlap = 8 bp). Next, both runs were merged by sample (resulting in 32 fastq format files), and checked for chimeras, using the DADA2 pipeline. Taxonomy was assigned to each Amplicon Sequence Variant (ASV) using the Silva small subunit rRNA database (v.132) with a minimum bootstrapping value of 80%. ASVs with non-bacterial origin (e.g., Archaea, chloroplast, mitochondria origin, and unclassified) were filtered out. ASVs with sequence length below 262 bp or above 292 bp were removed. The total survival following this step was 86.7 ± 7.9%. Then, a second filter was applied which removed all ASVs with a total read count <50. Overall, 32 chironomid samples resulted in 1,351,491 sequences (1,866–89,000 per sample), which were binned into 525 ASVs. To overcome the bias of unequal sequencing depth, we used ASV relative abundances per sample for downstream analysis.

The raw sequence data were deposited at the NCBI SRA repository^2^ under the accession number PRJNA634980.

### Statistical Analysis

We generated species accumulation curves, to evaluate if the sampling depth is sufficient to cover species richness, and calculated diversity indices of the bacterial communities using PRIMER v.7 (Plymouth Routines In Multivariate Ecological Research; Clarke and Gorley, 2015). Rarefaction curves and alpha diversity were calculated with PAST (Hammer et al., 2001). The richness (Chao1) and diversity (Shannon H') parameters were compared by Kruskal-Wallis tests, followed by Mann–Whitney *post-hoc* tests (IBM SPSS v.16.023). Analysis of similarities (ANOSIM) was applied to study the changes in bacterial community profiles between the different metamorphosis life stages. Differences between the microbiota compositions in the different life stages were analyzed by non-metric multidimensional scaling analysis (nMDS), and were based on both, Bray-Curtis and UniFrac (weighted and unweighted) distance matrices. The Bray-Curtis index nMDS was performed using PRIMER v.7 (Clarke and Gorley, 2015), while the UniFrac distance analysis was performed using the Microbial Analyst software (http://www.microbiomeanalyst.ca; Dhariwal et al., 2017). ASVs that were uniquely present in each life stage or shared between life stages were assessed using the InteractiVenn online tool (Heberle et al., 2015). The contribution of each variable or species to a Bray-Curtis dissimilarity matrix was calculated using similarity percentage (SIMPER).

## Results

The bacterial community composition of the four life stages of *C. transvaalensis*: egg masses, larvae, pupae, and adults (Supplementary Table 1), were studied using Illumina sequencing of the 16SrRNA gene. Rarefaction curves generated for the samples showed that sequencing depth was sufficient to encompass bacterial species richness. The species accumulation curves reached an asymptote after ~2,000 reads, indicating that the sequencing efforts were sufficient to obtain an accurate estimate of ASV richness (Supplementary Figure 1). The calculated species accumulation indices reached an asymptote after ~27 samples, indicating that the number of samples detected most bacterial genera (Supplementary Figure 2).

### The Effect of Life Stage on the Microbiota Composition and Diversity

#### Alpha Diversity

**Figure 1:**
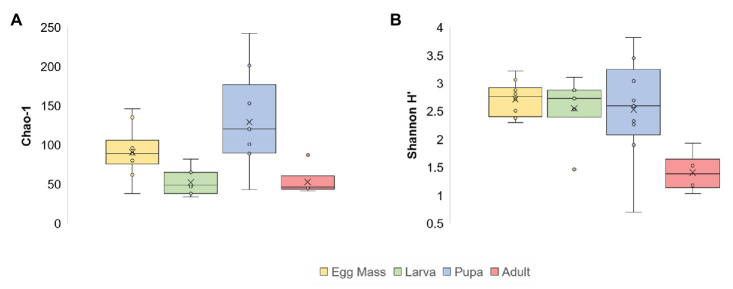
Alpha diversity of *C. transvaalensis* microbiota for each metamorphosis life stage [at the Amplicon Sequence Variant (ASV) level]. **(A)** Chao1 richness estimator; **(B)** Shannon H' Index. Significant differences were found between the microbiota richness of the four life stages (Kruskal-Wallis: *X*^2^_3_ = 14.805, *p* = 0.002) and specifically between the pupae and the larvae and the pupae and the adults (Mann–Whitney: *p* = 0.003; *p* = 0.0076, respectively). The Shannon H' Index was significantly different between the four life stages (Kruskal-Wallis: *X*^2^_3_ = 11.245, *p* = 0.01) and specifically between the egg masses and the adults and the larvae and the adults (Mann–Whitney: *p* = 0.00025; *p* = 0.0081, respectively). Egg masses: yellow; larvae: green; pupae: blue; adults: red.

Significant differences were found between the microbial richness (Chao1) of the four life stages (Kruskal-Wallis: *X*^2^_3_ = 14.805, *p* = 0.002). Richness was the highest in the pupal stage (1.4 times greater compared to the egg masses and 2.4 times more than the richness in the larvae and adults; Figure 1; Supplementary Table 2). Richness was significantly different between the pupae and larvae and the pupae and adults (Mann–Whitney: *p* = 0.003; *p* = 0.0076, respectively). When the bacterial diversity at the ASV level (Shannon H') was compared between the four metamorphosis life stages, significant differences were observed (Kruskal-Wallis: *X*^2^_3_ = 11.245, *p* = 0.01), most notably between the egg masses and adults (Mann–Whitney: *p* = 0.00025) and the larvae and adults (Mann–Whitney: *p* = 0.0081; Figure 1; Supplementary Table 2). In addition, the phylum diversity (Shannon H') was significantly lower in the adult stage than the other life stages (Kruskal-Wallis: *X*^2^_3_ = 9.53, *p* = 0.023), with the values of 1.2, 0.66, 0.5, and 0.25 for the larvae, pupae, egg masses, and adults, respectively (Supplementary Table 2).

#### Beta Diversity

**Figure 2:**
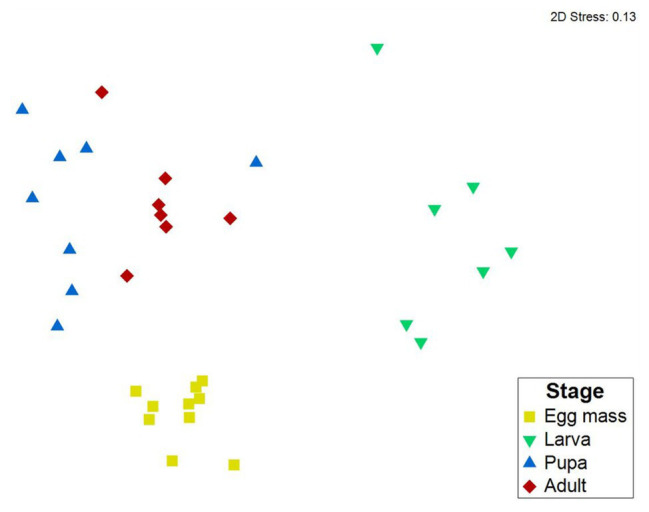
Non-metric multidimensional scaling (nMDS) analysis representing bacterial compositions in the four metamorphosis life stages of *C. transvaalensis*. Stress = 0.13 (Bray–Curtis), *n* = 32 samples. Significant differences were found between the microbiota compositions of all four life stages (ANOSIM: *R* = 0.874, *p* < 0.001). Egg masses: yellow; larvae: green; pupae: blue; adults: red.

Differences between *C. transvaalensis* bacterial community compositions in the different metamorphosis life stages were evaluated by nMDS ordination analysis and tested for significance with ANOSIM. The nMDS plot (Bray-Curtis dissimilarity matrix, stress = 0.13) showed that the microbiota composition of each life stage clustered separately (Figure 2). Significant differences were found between the microbiota compositions of the four life stages of *C. transvaalensis* (ANOSIM: *R* = 0.874, *p* < 0.001; Figure 2). Similar results were obtained when we performed nMDS analysis based on UniFrac distance matrices (ANOSIM: *R* = 0.55, *p* < 0.001; *R* = 0.67, *p* < 0.001 for the weighted and unweighted, respectively; Supplementary Figure 3). SIMPER analysis showed differences in the microbiota composition mainly between the larvae and pupae and between the larvae and adults (with 90.83 and 90.25% dissimilarities, respectively). Less dissimilarities were found between the adults and pupae and the adults and egg masses (with 72.77 and 79.62% dissimilarities, respectively).

### Taxa Composition in the Different Life Stages

**Figure 3:**
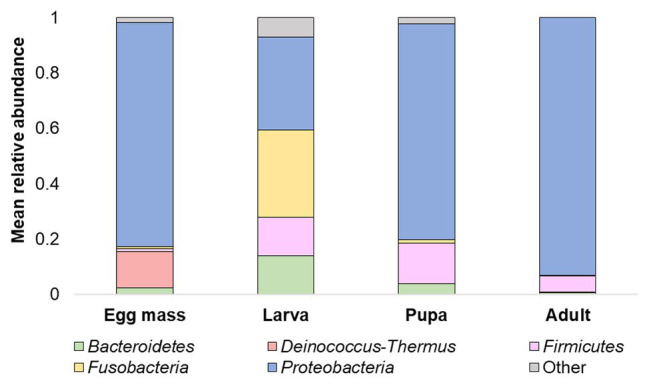
Mean relative abundance (>5%) of *C. transvaalensis* microbiota at the phylum level for each metamorphosis life stage.

**Figure 4:**
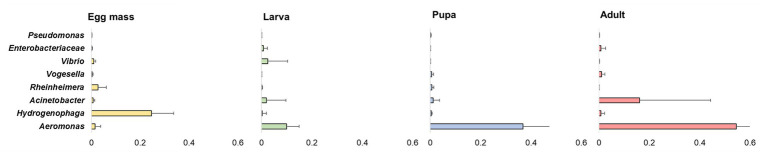
The average relative abundance of eight prominent genera identified in ≥75% of all samples (defined as core bacteria). Bars represent means ± SE.

Overall, 20 phyla were detected. The five most abundant (over 5% of the reads) across all samples were: *Proteobacteria*, *Firmicutes*, *Bacteroidetes*, *Fusobacteria*, and *Deinococcus-Thermus* (Figure 3). At the phyla and the ASV levels, significant differences were found between the bacterial communities of all the studied life stages of *C. transvaalensis* except between the pupae and the adults (ANOSIM: phyla: *R* = 0.467, *p* < 0.001; ASV: *R* = 0.874 *p* < 0.001; Figure 3). Overall, 151 genera were detected, of which 27 made up the bacterial core. This core was defined as the genera that were present in ≥75% of all samples. *Aeromonas* was the most dominant genus in the larva, pupa, and the adult life stages, while *Hydrogenophaga* was the most dominant genus in the egg mass stage (Figure 4). Other genera from the core bacteria that were also found in relatively high abundances in the different life stages were *Rheinheimera* (egg mass), *Vibrio* (larva), and *Acinetobacter* (adult; Figure 4). Molecular detection of the *ompW* gene provided evidence that the *Vibrio* ASVs represent *V. cholerae*, which was present in all the life stages of *C. transvaalensis* and in 78.1% (25/32) of the samples (Table 1).

### Venn Diagram

**Figure 5:**
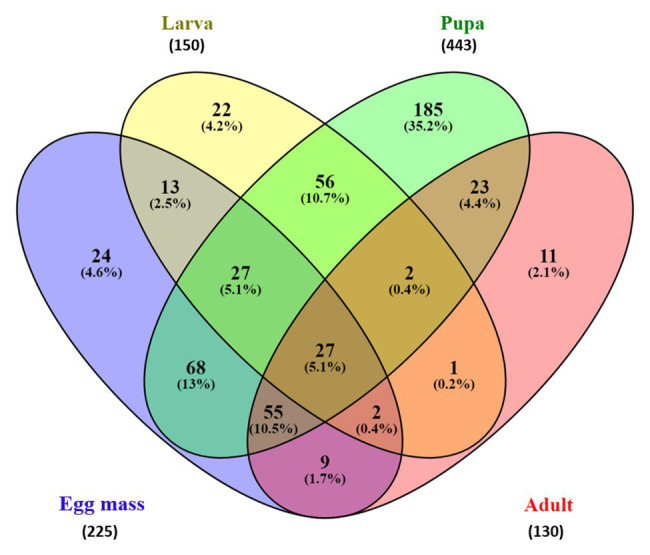
Venn diagram showing the limited overlap of *C. transvalensis* bacterial ASVs across the different metamorphosis life stages. The Venn diagram shows the percentage of ASVs that are uniquely present in each of the life stages and shared between pairs and groups of life stages. Egg masses: blue; larvae: yellow; pupae: green; adults: red.

The Venn diagram (Figure 5) which represents the shared and unique ASVs between each life stage, demonstrated a minor overlap between ASVs of the different metamorphosis stages. Only 27 ASVs were shared between all life stages, representing 5.14% of all the ASVs (Figure 5). Interestingly, pupa samples contained 443 ASVs, which is two times higher than the ASVs represented in the egg mass samples (225 ASVs) and three times higher than those found in the larva and the adult microbiota samples (150 and 130 ASVs, respectively). Moreover, the pupa samples had the largest portion of unique ASVs (35.2%, 185 ASVs) compared to the egg mass, larva, and adult samples (4.6, 4.2, and 2.1%, respectively). These correspond with the results of the pupal richness that was the highest compared to the other life stages (129.11 ± 61.18; Figure 1; Supplementary Table 2).

## Discussion

We have studied the change in the endogenous microbiota composition throughout the metamorphosis life stages of *C. transvaalensis*. To the best of our knowledge, this is the first study that explores the bacterial community in different developmental life stages of any chironomid species. Significant differences were found between the microbiota compositions of the four developmental life stages (Figure 2; Supplementary Figure 3). Similarly, significant differences were also demonstrated between the microbiota compositions of the different metamorphosis life stages of the moth *Brithys crini*, the butterfly *Heliconius erato*, and the ant *Atta* (Hammer et al., 2014; Zhukova et al., 2017; González-Serrano et al., 2019). The distinct bacterial communities in *H. erato* life stages were explained by the specific diets in each of its life stages, while the differences between the microbiota compositions of the larvae and the adults of the ant *Atta* were explained by the social behavior of the adult workers (Zhukova et al., 2017). A possible explanation for the differences between the microbiota compositions in the current study may stem from the different habitats encountered in the different stages: (i) water surface (egg masses), (ii) water sediment inside tubes built from silt and silk-like salivary secretion (larvae), (iii) water sediment inside the tubes and then on the water’s surface (pupae), and (iv) in the air (adults). The differences in the microbiota composition may also be because only the larval stage feeds (Armitage et al., 1995).

A decline in bacterial diversity at the ASVs level (Figure 1; Supplementary Table 2) was observed as the insect evolved from egg masses to adults, while the highest richness was observed in the pupae. The pupa is a transitional stage between life in water and terrestrial life. The larva transforms into pupa in sediment inside a tube and then, when the pupa is mature, it moves to the water surface and the adult emerges (Berg, 1995). This stage is critical in the chironomid life cycle because the pupa has two different habitats, and therefore, it may need a more diverse and richer microbiome for protection (Halpern and Senderovich, 2015). Similarly, a decrease in the bacterial diversity between the larval and the adult stages was observed in the moth *B. crini*. *B. crini* is a holometabolous insect and the larvae feed on *Pancratium maritimum*, a plant that produces toxic compounds. The authors hypothesized that the restricted larval diet requires a diverse microbiota that enables digestion of this plants’ specific compounds. They further explained that the bacterial diversity decreased in the adult stage as a result of changes in the adult intestine (González-Serrano et al., 2019). In *Culex tarsalis*, the bacterial community of the early instar larvae was significantly more diverse compared to the other life stages (late instar larvae, pupae, and adults; Duguma et al., 2015). Studies of several *Anopheles* species found that the richness and the bacterial diversity in the adult stage were lower compared to the larval and the pupal stages (Wang et al., 2011; Gimonneau et al., 2014; Bascuñán et al., 2018). *Chironomus*, *Culex*, and *Anopheles* metamorphosis include three life stages in the water and a terrestrial adult stage; thus, it may explain why their diversity pattern is similar.

It was thought that metamorphosis that occurs in mosquitoes eliminate most of the microbial communities; however, evidence of bacteria that were transmitted from one developmental stage of the host to its subsequent stage (transstadial transmission), suggested that bacterial clearance is not complete during life development (Alfano et al., 2019). We found that as the insect develops, it transfers bacterial ASVs to the next level. About 36% of the bacterial ASVs in the mosquito *Aedes koreicus* were shared between the larva and the pupa, and 24% of those observed in the pupa were found in the adult. These changes were explained as the renewal of the intestinal epithelial layers that occurs during the mosquito metamorphosis development (Alfano et al., 2019). In our study, 13.1% of the ASVs were shared between the egg masses and the larvae. This may be explained by the fact that the first instar larva feeds on the egg mass gelatinous matrix. Then, as the larva develops to the next instar stage, it swims to the bottom of the water habitat; builds a silken tube; and feeds on algae, detritus, and associated microorganisms, macrophytes, wood debris, and invertebrates (Berg, 1995). Overall, 112 ASVs (21.3%) were shared between the larvae and the pupae, and 107 ASVs (20.4%) were shared between the pupa to the adult stage. Ninety-three ASVs (17.7%) were shared between the adult and the egg mass stages (Figure 5).

The Venn diagram of shared ASVs between the different life stages in this study illustrates that while each of the metamorphosis life stages had unique ASVs, there are still shared ASVs between the life stages (Figure 5). In the current study, only 27 ASVs (5.14%) were shared between all the life stages and belonged to the core microbiota (detected in ≥75% of the sample). Interestingly, most of the ASVs in the core belonged to the *Proteobacteria* phylum. *Proteobacteria* species are known as primary insect symbionts and as one of the most abundant phyla in insects (Jones et al., 2013). Low overlapping of bacterial ASVs was observed between the life stages of the moth *B. crini* and most of them belonged to *Proteobacteria* (González-Serrano et al., 2019). Similarly, few shared operational taxonomic units (only 2%) were found in the mosquito *C. tarsalis* (Duguma et al., 2015).

*Proteobacteria*, *Firmicutes*, and *Bacteroidetes* were the dominant phyla in the current study (Figure 3) as well as in previous studies of chironomid egg masses and larvae microbiota composition (Senderovich and Halpern, 2013; Sela and Halpern, 2019). Nevertheless, in the current study, *Deinococcus-Thermus* was a dominant phylum in the egg masses (Figure 3) and was never described in chironomids before. This phylum contains extremophilic bacterial species that are resistant to radiation and extreme temperatures (Ho et al., 2016). Chironomids lay their egg masses on the water’s edge, and in the summertime, the egg masses are exposed to high temperatures and UV radiation. Chironomids are known to be temperature‐ and UV radiation-tolerant (Thorat and Nath, 2015, 2016). Thus, *Deinococcus-Thermus* bacterial species may play a role in protecting the egg masses from extreme conditions; however, the direct effect of *Deinococcus-Thermus* on chironomid survival under high temperature and UV radiation has not yet been studied.

*Rheinheimera* was one of the core bacteria in the current study and was relatively more abundant in the egg mass stage (Figure 4). *Rheinheimera* was also previously identified in *C. transvaalensis* egg mass microbiota (Senderovich and Halpern, 2013; Sela and Halpern, 2019). Species of this genus have biodegradation abilities (Virk et al., 2012). *Hydrogenophaga*, *Acinetobacter*, and *Pseudomonas* were also identified in the current study as core bacteria (Figure 4). *Hydrogenophaga* was relatively more abundant in the egg mass stage, while *Acinetobacter* was relatively more abundant in the adult stage. Species of these genera were previously detected in *C. transvaalensis* egg masses microbiota and were suggested to play a role in protecting the insect from toxic compounds (Ali, 1991; Senderovich and Halpern, 2012, 2013; Sela and Halpern, 2019).

*Vibrio*, detected in all four life stages of *C. transvaalensis*, was also one of the genera in the core bacteria (Figure 4). The abundance of *Vibrio* ASVs was higher in larvae compared to the other life stages (Figure 4). Halpern et al. (2007), quantified viable *V. cholerae* cells in freshly collected egg masses using fluorescence *in situ* hybridization (FISH) technique. An average of 3.9 × 10^4^ viable *V. cholerae* cells were counted per egg mass. In the current study, the PCR limit of detection for *ompW* gene was found to be about 9.3 × 10^3^ *V. cholerae* cells per specimen (unpublished data), indicating that each *ompW* PCR positive sample inhabits at least about 10^4^ *V. cholerae* cells per sampled specimen (egg mass, larva, pupa or adult).

*Vibrio cholerae*, a human pathogen that causes the diarrheal cholera disease is a natural inhabitant of aquatic ecosystems and is commonly isolated along with other bacteria from all four chironomid life stages (Halpern and Senderovich, 2015). *ompW* gene (an outer membrane protein specific for *V. cholerae*), was detected in most *C. transvaalensis* samples, demonstrating that *V. cholerae* inhabits all life stages of the insect (Table 1). Hence, this shows that *C. transvaalensis* is a host of *V. cholerae*. Using an experimental approach, Broza et al. (2005), demonstrated that *V. cholerae* O1 can be transferred from infected larvae to emerging adults. Furthermore, in a field experiment, they showed that chironomid flying adults can infest clean water pools with environmental *V. cholerae*. They concluded that chironomid adults can disperse *V. cholerae* between water bodies (Broza et al., 2005). Similarly, Dickson et al. (2017), found that larval exposure to pathogenic bacteria during the developmental stage can affect the ability of flying mosquito adults to transmit pathogens (Dickson et al., 2017).

*Aeromonas* ASVs were also detected in all four life stages of *C. transvaalensis*. This genus was a part of the core bacteria (Figure 4). The following *Aeromonas* species were found to inhabit chironomid egg masses: *Aeromonas caviae* (*punctata*), *Aeromonas culicicola*, *Aeromonas dhakensis* (formerly *aquariorum*), *Aeromonas hydrophila*, *Aeromonas media*, *Aeromonas salmonicida*, *Aeromonas sanarellii*, *Aeromonas schubertii*, *Aeromonas taiwanensis*, and *Aeromonas veronii* (Laviad and Halpern, 2016). In the current study, the lowest *Aeromonas* prevalence were observed in the egg masses, and it gradually increased through the larva and pupa stages to the adult stage which hosted the highest prevalence of this genus (54% ± 0.25) (Figure 4). The results of the current study support previous evidence suggesting that chironomids are reservoirs of *Aeromonas* species (Senderovich et al., 2008; Laviad and Halpern, 2016). Chironomid species can survive in toxic environments (Halpern and Senderovich, 2015), and *Aeromonas* species were found to protect chironomid larvae from toxic metals (Senderovich and Halpern, 2012, 2013).

It was shown that both *V. cholerae* and *Aeromonas* species can degrade the chironomid egg mass’s gelatinous matrix, consisting of glycoprotein and chitin (Halpern et al., 2003; Laviad et al., 2016). *Vibrio cholerae* degrades the egg masses’ glycoprotein matrix by secreting haemagglutinin protease, while *Aeromonas* degrades chitin by secreting chitinases. This egg mass degradation prevents larval hatching and thus, controls chironomid population levels on the one hand (Halpern, 2010) and may support the maintenance of the egg mass’s endogenous bacterial communities, on the other hand, by supplying them with available nutrients (Laviad et al., 2016; Sela et al., 2020). Thus, the presence of *V. cholerae* and *Aeromonas* species in all four life stages of *C. transvaalensis* might represent a mutualistic relationship between these species and their natural host. Jones et al. (2013), showed that host-symbionts can have a large influence on insect hosts, often more than other factors, including diet, distribution, distant, and more ecological factors (Jones et al., 2013). We propose that *V. cholerae* and *Aeromonas* species are *C. transvaalensis* symbionts.

## To Conclude

The microbiota composition of *C. transvaalensis* changed across its four metamorphosis developmental life stages. As far as we know, this is the first study that explored the dynamics of bacterial communities in any chironomid species. The bacterial community composition that is specific to each developmental life stage, likely plays a role in promoting the insect’s development and survival. However, these relationships still must be proven by inducing dysbiosis in *C. transvaalensis* egg masses or larvae and then following the developmental process of the insect. A failure to complete a healthy metamorphosis life cycle will provide support that the endogenous microbiota of each life stage plays a crucial part in *C. transvaalensis* development.

## Data Availability Statement

The nucleotide sequence data reported are available in the NCBI GenBank database under the accession number(s): MN934105-MN934150 and RPJNA634980.

## Author Contributions

RS, SL-S, and MH conceived and designed the experiments. MH contributed reagents, materials and analysis tools. SL-S performed the experiments. RS analyzed the data and wrote the paper. SL-S and MH reviewed and commented the paper. All authors contributed to the article and approved the submitted version.

### Conflict of Interest

The authors declare that the research was conducted in the absence of any commercial or financial relationships that could be construed as a potential conflict of interest.

## Footnotes

^1^https://www.bioinformatics.babraham.ac.uk/projects/fastqc/

^2^https://www.ncbi.nlm.nih.gov/sra
