# Supplementary material for: Changes in Microbiota Composition Along the Metamorphosis Developmental Stages of Chironomus transvaalensis
Source: Front Microbiol. 2020 Nov 6;11:586678. doi: 10.3389/fmicb.2020.586678 (PMC7677345; doi:10.3389/fmicb.2020.586678)
Supplement: Supplementary file 1 [file Data_Sheet_1.pdf]

Supplementary Material

Supplementary Figures and Tables

Supplementary Tables

Supplementary Table 1. A list of *C. transvaalensis* samples that were analyzed in the current study. All four life stages of the insect were sampled on the same day from the Yokneam waste stabilization pond in northern Israel (July 2018).

**Egg masses**	**Larvae**	**Pupae**	**Adults**
YEM1	YSL2	YP1	YA1
YEM2	YSL3	YP3	YA2
YEM3	YSL4	YP4	YA6
YEM4	YSL6	YP5	YA7
YEM5	YSL8	YP6	YA8
YEM6	YBL5	YP7	YA9
YEM7	YBL9	YP9	
YEM8		YP10	
YEM9		YP11	
YEM10			
**Total** (32)			
10	7	9	6

*Y, Yokneam waste stabilization pond; EM, Egg mass; SL and BL, Larva; P, Pupa; A, adult.

Supplementary Table 2. Estimated richness and diversity indices for each life stage of *C. transvaalensis* at the ASV and the phylum levels.

**Taxonomy level**	**Richness** (Chao1)<br>Avg ± SE	**Diversity** (Shannon H')<br>Avg ± SE
**ASV**		
Egg masses	90.6 ± 31.38	2.71 ± 0.31
Larvae	52.14 ± 16.46	2.55 ± 0.53
Pupae	129.11 ± 61.18	2.53 ± 0.91
Adults	52.83 ± 17.10	1.41 ± 0.33
**Phylum**		
Egg masses	9.10 ± 0.48	0.50 ± 0.08
Larvae	7.71 ± 0.56	1.21 ± 0.62
Pupae	9.67 ± 1.25	0.66 ± 0.10
Adults	5.83 ± 0.60	0.25 ± 0.48

Shannon H' and Chao1 indices are given for each sampling stage. Avg, average; SE, standard error.

Supplementary Figures

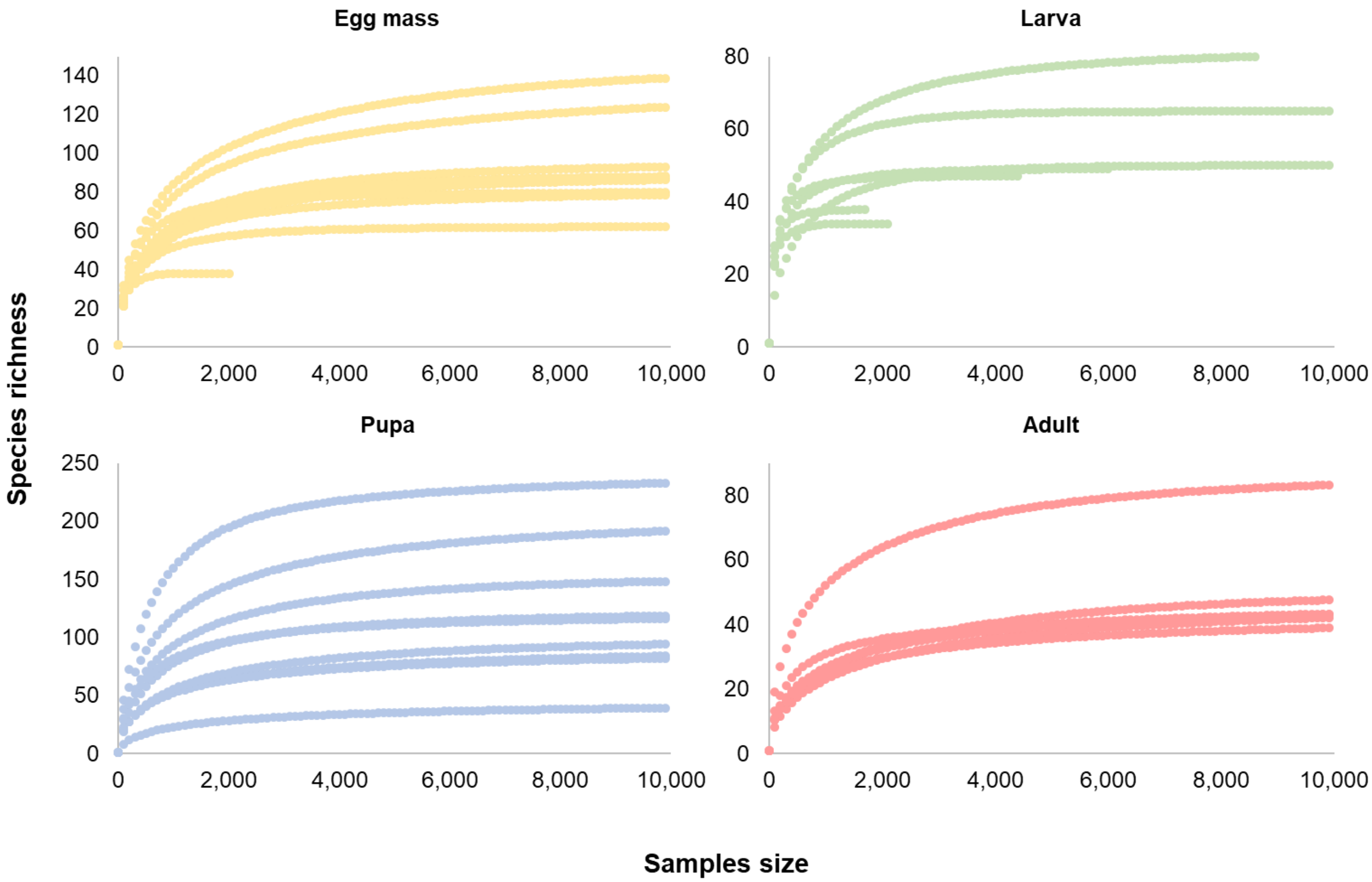

Supplementary Figure 1. **Rarefaction curves indicating the observed number of Amplicon Sequence Variants (ASVs) in all chironomid samples.** The rarefaction curves represent the increase in the number of ASVs as a function of the sequences number of each individual sample.

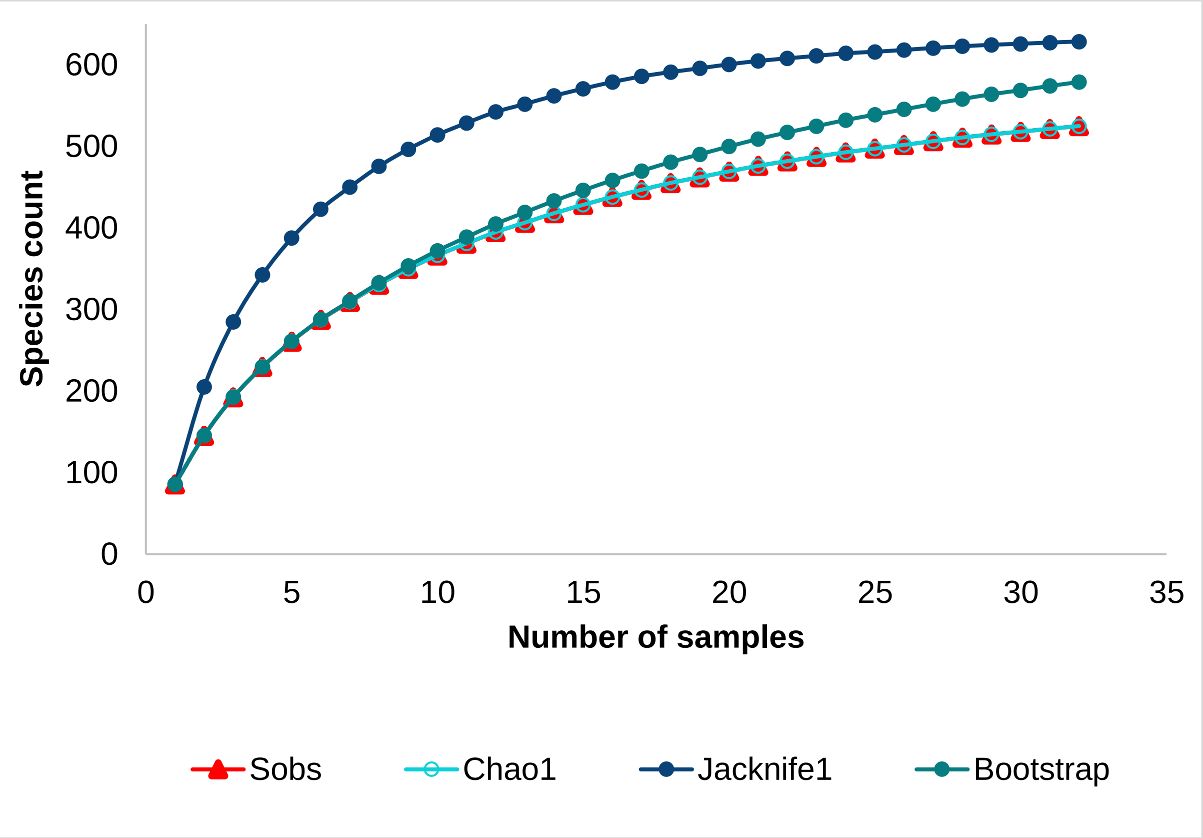

Supplementary Figure 2. Species accumulation curves and richness indices of the bacterial communities inhabiting the four metamorphosis life stages of *C. transvaalensis*. Red triangles represent the observed accumulated species richness (Sobs). Light blue hollow circles represent the abundance-based estimator of species richness (Chao1). Blue circles represent presence or absence-based estimator of species richness (Jacknife1). Turquoise circles represent an estimate of true richness (Bootstrap).

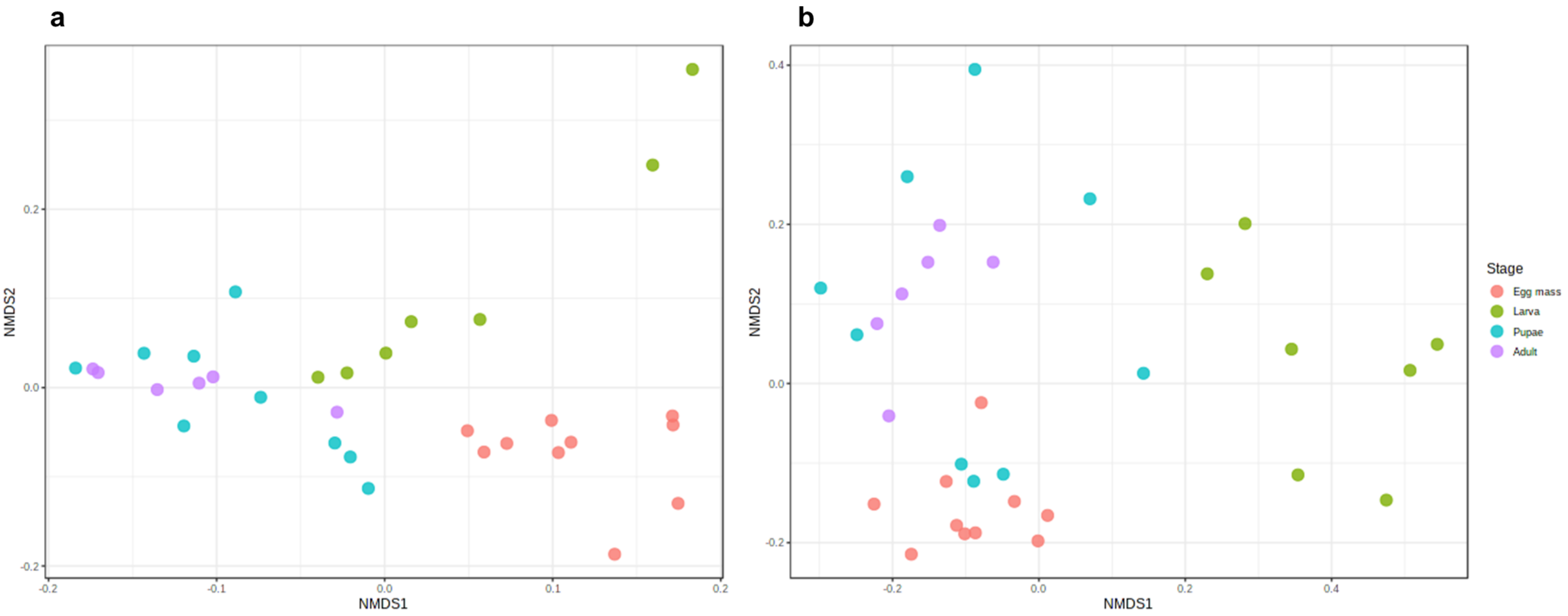

Supplementary Figure 3. nMDS based on the weighted (a) and unweighted (b) UniFrac distance matrices estimated from the bacterial composition of the four metamorphosis life stages of *C. transvaalensis*. a. Weighted UniFrac, stress = 0.11, n = 32. **b.** Unweighted UniFrac, stress = 0.13, n = 32. Significant differences were found between all the microbiota compositions of all four life stages (ANOSIM: R = 0.55, $p < 0.001$; R = 0.67, $p < 0.001$ for the weighted and unweighted, respectively).
